# Supplementary material for: Mineral Content and Volatile Profiling of Prunus avium L. (Sweet Cherry) By-Products from Fundão Region (Portugal)
Source: Foods. 2022 Mar 4;11(5):751. doi: 10.3390/foods11050751 (PMC8909425; doi:10.3390/foods11050751)
Supplement: Supplementary file 1 [file foods-11-00751-s001.zip › foods-1599834-supplementary.pdf]

Table S1. Limits of detection (LOD) and limits of quantification (LOQs) for the analyzed elements.

Element	LOD (μg/L)	LOQ (μg/L)
Al	1.92	6.40
As	0.033	1.09
Ba	0.05	0.17
Cd	0.03	0.10
Co	0.04	0.15
Cr	0.14	0.45
Cu	0.42	1.39
Fe	11.04	36.81
Li	0.01	0.03
Mn	0.06	0.21
Na	23.08	76.86
Ni	0.87	2.90
Pb	0.06	0.21
Rb	0.13	0.42
Se	0.25	0.84
Sr	0.10	0.33
Zn	0.79	2.61
